# Hippocampal Subfields and White Matter Connectivity in Patients with Subclinical Geriatric Depression

**DOI:** 10.3390/brainsci12030329

**Published:** 2022-02-28

**Authors:** Jeonghwan Lee, Gawon Ju, Hyemi Park, Seungwon Chung, Jung-Woo Son, Chul-Jin Shin, Sang Ick Lee, Siekyeong Kim

**Affiliations:** 1 Department of Psychiatry, Chungbuk National University Hospital, Cheongju 28644, Korea; jeonghwanmail@gmail.com (J.L.); baugita@gmail.com (G.J.); ideogrami@naver.com (H.P.); shanelk@hanmail.net (S.C.); mammosss@hanmail.net (J.-W.S.); cjshin@gmail.com (C.-J.S.); silee@chungbuk.ac.kr (S.I.L.); 2 Department of Psychiatry, College of Medicine, Chungbuk National University, Cheongju 28644, Korea

**Keywords:** geriatric psychiatry, depression, hippocampus, memory, diffusion tensor imaging

## Abstract

Despite an abundance of research related to the functional and structural changes of the brain in patients with geriatric depression, knowledge related to early alterations such as decreased white matter connectivity and their association with cognitive decline remains lacking. We aimed to investigate early alterations in hippocampal microstructure and identify their associations with memory function in geriatric patients with subclinical depression. Nineteen participants with subclinical geriatric depression and 19 healthy controls aged ≥65 years exhibiting general cognitive function within the normal range were included in the study and underwent assessments of verbal memory. Hippocampal subfield volumes were determined based on T1-weighted magnetization-prepared rapid gradient echo (T1-MPRAGE) images, while group tractography and connectometry analyses were conducted using diffusion tensor images. Our findings indicated that the volumes of whole bilateral hippocampus, cornus ammonis (CA) 1, molecular layer, left subiculum, CA3, hippocampal tail, right CA4, and granule cell/molecular layers of the dentate gyrus (GC-ML-DG) were significantly smaller in the subclinical depression group than in the control group. In the subclinical depression group, verbal learning was positively correlated with the volumes of the CA1, GC-ML-DG, molecular layer, and whole hippocampus in the right hemisphere. The fractional anisotropy of the bilateral fornix was also significantly lower in the subclinical depression group and exhibited a positive correlation with verbal learning and recall in both groups. Our results suggest that hippocampal microstructure is disrupted and associated with memory in patients with subclinical depression.

## 1. Introduction

Geriatric depression is a common psychiatric disorder among older adults. Despite the approximately 1% prevalence of major depressive disorder in adults aged ≥65 years, 15% experience mild or moderate depressive symptoms not meeting the criteria for major depressive episodes [1]. Furthermore, research among the geriatric population has indicated that 20% of outpatients and 40% of inpatients experience depressive symptoms [2].

Patients with geriatric depression often do not directly report their depressive symptoms, but rather express concerns regarding cognitive decline, including decreased concentration, memory impairment, and slower processing speed. Therefore, specialized care must be provided to such patients, and there is a need to further investigate the relationship between depressive disorders and cognitive decline in older adults [3]. Pseudodementia due to depression can easily be mistaken for irreversible dementia, such as that associated with Alzheimer’s disease (AD) and other common age-related diseases, thus decreasing the likelihood of patients to receive appropriate treatment. Moreover, studies supporting the direct and indirect effects of depressive disorders on cognitive function in older adults highlight the importance of such relationships in the geriatric population [4]. For instance, four hypotheses have been proposed regarding the complex relationship between depression and dementia: (a) Depressive symptoms are prodromes of AD; (b) depression is a risk factor for AD; (c) depression and AD are independent of each other; and (d) depression and AD may develop independently, but depression affects the progression and treatment of cognitive impairment [5]. 

White matter hyperintensity, decreased hippocampal volume, and reduced blood flow are common in geriatric patients with cognitive impairment and depressive disorders [6,7,8,9,10]. A previous study has suggested an association between decreased hippocampal volume and depressive symptoms in older adults [11], which may explain the memory decline observed in patients with geriatric depressive disorder. In addition to hippocampal volume, interhemispheric hippocampal functional and structural connectivity have been associated with memory function in previous studies [12,13]. However, to our knowledge, no studies have investigated whether geriatric depression is associated with alterations in hippocampal structural connectivity. 

Since the clinical characteristics of geriatric depression differ from those of depression in younger adults, early screening and identification of changes are important in ensuring that patients receive appropriate treatment. In this study, we assessed changes in cognitive function and brain structure in older adults with symptoms of subclinical depression. We hypothesized that memory and hippocampal microstructure are disrupted and reciprocally associated older adults with subclinical depression. 

## 2. Materials and Methods

### 2.1. Participants and Neuropsychological Tests

This study included 38 adult participants (26 women, 12 men; age ≥ 65 years) residing in a senior welfare center located in Cheongju, Chungcheongbuk-do Province, South Korea. None of the participants had a diagnosis of major depressive disorder, schizophrenia, or other psychotic disorders; bipolar or related disorders; or substance-related/addictive disorders based on an interview with a psychiatrist using the Structured Clinical Interview of the DSM-IV (SCID). Participants with brain damage, those with neurological disorders including epilepsy, and those with other systemic disorders that may affect the central nervous system were excluded. 

Participants who scored ≥ 8 on the Korean Version of the short form of the Geriatric Depression Scale (GDS-K) were classified into the subclinical depression group [14], while those who scored < 8 were classified into the control group. Verbal memory was assessed using the word-list learning, word-list recall, and word-list recognition tests of the Korean Version of the Consortium to Establish a Registry for Alzheimer’s Disease (CERAD-K) assessment [15]. The general cognitive function of all participants was assessed using the Mini-Mental State Examination included in the Korean Version of the CERAD Assessment Packet (MMSE-KC) [15]. The MMSE-KC scores of all participants were above the –1-standard deviation (SD), in accordance with standardized normative data. All participants provided written informed consent, and the study was approved by the Institutional Review Board of Chungbuk National University (CBNU-201406-BMSBBR-059-01). 

### 2.2. Structural MRI Acquisition

Imaging data were acquired using the 3T Achieva MRI scanner (Phillips Medical Systems, Best, The Netherlands) at the Korea Basic Science Institute (Ochang, South Korea). T1-weighted magnetization-prepared rapid gradient echo (T1-MPRAGE) images were acquired using the following sequences: repetition time/echo time = 6.8/3.2 ms, flip angle = 9°, bandwidth = 241.1 Hz, field-of-view (FOV) = 256 × 240 mm^2^, voxel size = 1 × 1 × 1.2 mm^3^, scan time = 5 m 34 s, and 170 slices. Diffusion tensor images (DTIs) were acquired using the following sequences: repetition time/echo time = 6033/70 ms, flip angle = 90°, bandwidth = 29.8 Hz, FOV = 224 × 224 mm^2^, voxel size = 2 × 2.04 × 3 mm³, diffusion gradient pulse duration = 34.4 ms, diffusion gradient separation = 12.3 ms, b-value = 1000 s/mm², scan time = 3 m 31 s, and 50 slices.

### 2.3. Image Analysis

**Figure 1:**
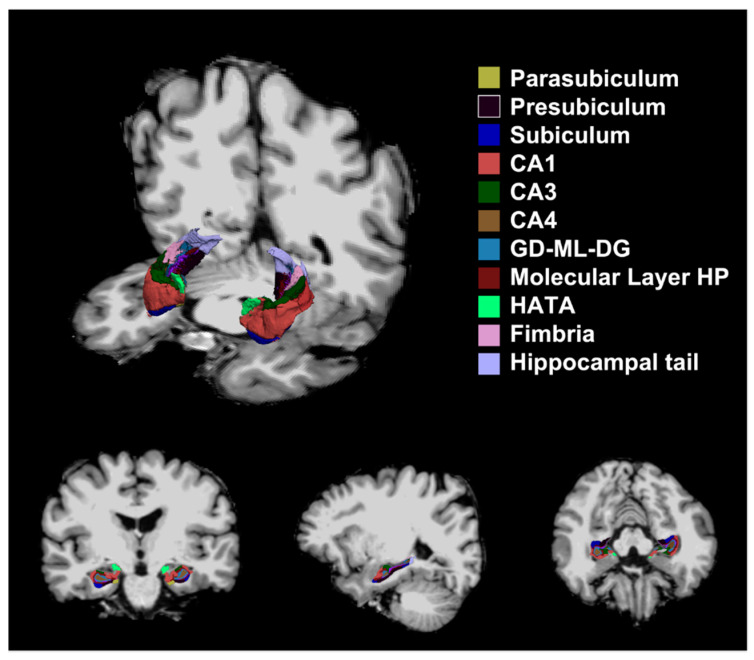
Segmentation of hippocampal subfields. CA: cornus ammonis, GC-ML-DG: granule cell and molecular layer of the dentate gyrus, HATA: hippocampus-amygdala-transition-area.

Hippocampal subfield volumes were quantified on T1-MPRAGE images via an automated method using FreeSurfer’s (version 7.1.1, http://surfer.nmr.mgh.harvard.edu, accessed on 28 February 2022) default settings on a Mac Pro (Apple, Cupertino, CA, USA) running the 64-bit OS X operating system [16]. We measured the volumes of the parasubiculum, presubiculum, subiculum, cornus ammonis (CA) 1, CA3, CA4, granule cell and molecular layers of the dentate gyrus (GC-ML-DG), molecular layer, hippocampus-amygdala-transition-area (HATA), fimbria, hippocampal tail, and hippocampal fissure (Figure 1). Volume measurements of hippocampal subfields underwent a correction process using the estimated total intracranial volume (eTIV) [17,18]. We carefully inspected the automatic hippocampal subfield segmentation results of all participants on axial, sagittal, coronal images referring the quality control protocol suggested by Samman et al., and confirmed that the segmentations were performed properly [19].

The white matter connectivity of the hippocampus was acquired based on DTIs using DSI studio [20]. DTIs were reconstructed using generalized Q-Space Diffeomorphic reconstruction (QSDR), which is suitable for analysis in Montreal Neurological Institute (MNI) space [21]. All R-squared values between quantitative anisotropy (QA) in the native space and the MNI QA map were above 0.6, implying good registration. A population average template of fractional anisotropy was then created using the QSDR files of all patients [22]. 

### 2.4. Statistical Analysis

The CRAN R statistical package version 3.6.2 (R Foundation for Statistical Computing, Vienna, Austria) was used for statistical analyses. Student’s *t*-test or Man-Whitney U-tests were used to compare demographic variables, GDS-K scores, MMSE-KC scores, and standardized scores on the word-list tests. Volumes of the hippocampal subfields were compared via an analysis of covariance (ANCOVA), using age as a covariate. We conducted Pearson’s correlation analysis between standardized scores on verbal memory tests and volumes of hippocampal microstructure. All *p*-values from hippocampal subfields were corrected for multiple comparisons using the false-discovery rate in the unilateral hemisphere. 

We utilized a correlation tractography and connectometry analysis tool in DSI studio to assess white matter connectivity [23]. On the population average template created through reconstruction of DTIs, the hippocampus was set as the seed region, while the cerebellum was excluded. Before comparing connectomety results using the hippocampus as the seed region, we conducted an exploratory analysis without using a seed region. Parameters for deterministic tractography and group connectometry after regressing out the effect of age were as follows: T-score threshold = 2.5, length thresholds = 20 mm, FDR threshold = 0.05, 4000 randomized permutations. We also assessed the association between scores on verbal memory tests and hippocampal white matter connectivity in each group with parameters equivalent to those mentioned for the group comparison above.

## 3. Results

### 3.1. Demographic Characteristics and Neuropsychological Functions in Each Group

There was no difference in mean age between the subclinical depression and control groups (72.37 ± 4.6 years and 69.58 ± 4.51 years; Table 1). The male-to-female ratio was equivalent between the groups, and there were no significant differences in years of education or eTIV. However, the mean GDS-K score was significantly higher in the subclinical depression group than in the control group (10.26 ± 2.10 vs. 2.21 ± 2.27, *p* < 0.001). Although there was no difference in MMSE-KC scores, the subclinical depression group performed significantly worse in the word-list recall (*p* = 0.014) and word-list recognition (*p* < 0.001) tests than the control group.

### 3.2. Hippocampal Microstructure

Bilateral hippocampal volumes were smaller in the subclinical depression group than in the control group (left: F = 8.52, corrected *p* = 0.020, right: F = 7.79, corrected *p* = 0.035; Table 2), as were the bilateral CA1 (left: F = 6.41, corrected *p* = 0.039, right: F = 6.36, corrected *p* = 0.042), molecular layer (left: F = 8.77, corrected *p* = 0.020, right: F = 8.08, corrected *p* = 0.035), left subiculum (F = 6.21, corrected *p* = 0.039), left CA3 (F = 9.44, corrected *p* = 0.020), left hippocampal tail (F = 15.10, corrected *p* < 0.001), and right CA4 volumes (F = 7.79, corrected *p* = 0.035) right GC-ML-DG (F = 6.35, corrected *p* = 0.042) (Table 2). 

**Figure 2:**
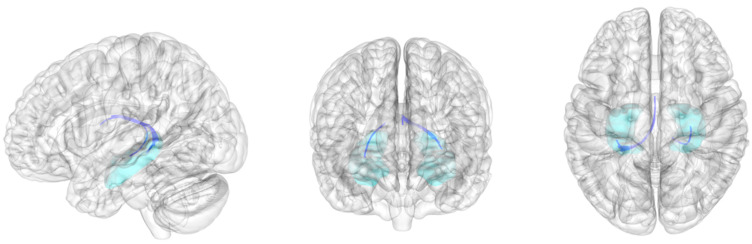
Group connectometry analysis. The blue indicates the fiber bundles exhibiting significantly decreased fractional anisotropy in the subclinical depression group when compared with that in the control group after correction for multiple comparisons (false-discovery rate < 0.001). The fiber bundles are part of the bilateral fornix. The cyan shading indicates the hippocampus.

In the exploratory analysis of whole brain connectometry, the majority of bilateral fornix fiber bundles exhibited lower fractional anisotropy in the subclinical depression group (Figure S1). Connectometry analysis using the hippocampus as the seed region also indicated that the fractional anisotropy of bilateral fornix was significantly lower in the subclinical depression group than in the control group (FDR < 0.001) (Figure 2).

### 3.3. Association between Hippocampal Microstructure and Verbal Memory

**Figure 3:**
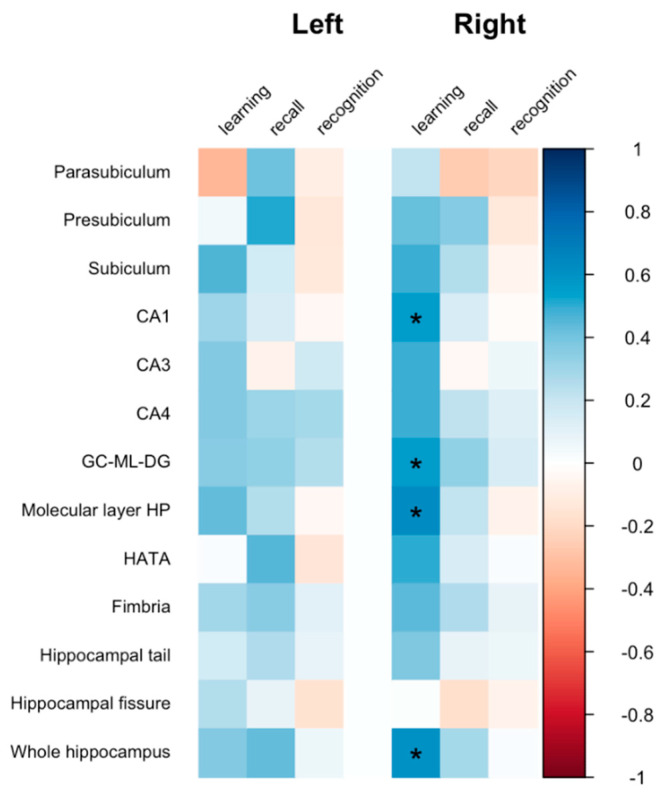
Correlation between hippocampal subfield volume and memory score.* *p* < 0.05 after correction for multiple comparisons using the false-discovery rate. The color scale bar represents the Pearson’s correlation coefficient. CA: cornus ammonis, GC-ML-DG: granule cell and molecular layer of the dentate gyrus, HATA: hippocampus-amygdala-transition-area.

Standardized scores on the word-list learning test were positively correlated with the volumes of the CA1 (cor = 0.563, corrected *p* = 0.043), GC-ML-DG (cor = 0.560, corrected *p* = 0.042), molecular layer (cor = 0.627, corrected *p* = 0.039), and whole hippocampus (cor = 0.605, corrected *p* = 0.039) of the right hemisphere in the subclinical depression group (Figure 3). However, there were no significant associations in the control group. The analysis of connectometry data indicated that the fractional anisotropy of the bilateral fornix fiber bundle was positively correlated with word-list memory and recall test results in both groups (FDR < 0.001) (Figure S2).

## 4. Discussion

To the best of our knowledge, this is the first study to investigate hippocampal microstructure, including hippocampal white matter connectivity, in older adults with subclinical depression using tractography and connectometry. Although all participants in the present study exhibited normal general cognitive function, there were significant impairments in verbal recall and recognition in the subclinical depression group. Marked volumetric reductions in the hippocampus and hippocampal subfields and disrupted integrity of fornix were also observed in the subclinical depression group. 

In this study, we observed volumetric reductions in hippocampal subfields associated with mood regulation and anxiety, including the bilateral CA1, molecular layer, left subiculum, CA3, hippocampal tail, right CA4, and GC-ML-DG. Another study including patients with chronic headache reported that higher levels of anxiety were associated with volumetric changes in the molecular layer, CA4, and GC-ML-DG [24]. Our findings are also in accordance with another study of geriatric depression, which reported reduced CA1 and subiculum volumes in the depression group [25]. The CA1, which is the first region of hippocampal circuit, projects to the subiculum, which in turn projects to brain structures associated with mood regulation, including the entorhinal cortex, amygdala, ventromedial prefrontal cortex, and corpus striatum. At the same time, the subiculum is also connected to brainstem nuclei associated with homeostatic networks, such as the hypothalamic–pituitary–adrenal axis. Studies have demonstrated that damage to this axis can lead to depressive disorders chronic abnormal secretion of stress hormones, and hippocampal atrophy [26,27]. 

In the exploratory analysis conducted at the whole-brain level, the majority of bilateral fornix fiber bundles exhibited reduced fractional anisotropy, suggesting a disruption in the structural integrity of the white matter connections (e.g., demyelination) [28,29]. In the hippocampal seed-based analysis, the fractional anisotropy of the bilateral fornix was significantly decreased in the subclinical depression group, while it was positively correlated with verbal learning and recall in both groups. The fornix plays a key role in hippocampal output and exhibits connections with the limbic system and the opposite hemisphere [30]. Wang et al. reported that interhemispheric hippocampal functional connectivity is associated with the recall of recently learned information [12], while another study also reported that recognition memory was correlated with the mean diffusivity of the dorsal hippocampal commissure, which is the white matter tract connecting the bilateral hippocampi [13]. Further, the absence of the hippocampal commissure has been associated with impaired long-term and short-term memory in an animal model [31]. In the subclinical depression group, verbal learning was positively correlated with the volumes of several right hippocampal subfields (including CA1, GC-ML-DG, the molecular layer) and the whole hippocampus, which were significantly smaller than those in the control group. On the contrary, none of the hippocampal subfield volumes were correlated with scores on the word-list memory test in the control group. Our results suggest that volumetric reductions and disrupted integrity in hippocampal microstructures are associated with memory impairment in patients with subclinical geriatric depression.

The present study has a few limitations. First, we recruited only a small number of participants which might not be sufficient for conclusive results. Also, we applied FDR method for multiple comparison correction in unilateral hemisphere to avoid chance of type II error. Therefore, our study results should be interpreted cautiously, and further studies with larger sample sizes are needed to confirm our findings. Second, we included participants with subclinical geriatric depression. Therefore, several factors related to depressive symptoms may not have been controlled. For example, we may have included patients with age-related depressive symptoms tolerated even beyond the normal range. Nonetheless, we recruited socially active community members while excluding individuals with other neuropsychiatric disorders using structured psychiatric interviews, and the study was conducted under the assumption that patients were in the prodromal stage or at risk of geriatric depression. Lastly, as this was a cross-sectional study, we were unable to determine whether the observed differences in brain structure between groups were due to subclinical depression, or whether depression was a result of structural changes in the brain. However, similar previous studies, including longitudinal investigations, have suggested that brain abnormalities are a predisposing factor for the development of depressive disorders [9]. Thus, the differences observed in the present study may reflect predisposition or early changes at a subclinical level. These data may aid in elucidating the etiology of geriatric depression. 

Despite these limitations, our analysis identified disruptions of the hippocampal microstructures and their association with memory impairment in participants with subclinical geriatric depression. Although the findings should be interpreted with caution, they may provide insight into signs of cognitive decline and emotional symptoms experienced by individuals with subclinical geriatric depression.

## Figures and Tables

**Table 1:** Demographic characteristics and memory scores in each group (mean ± SD).

	Subclinical Depression (n = 19)	Control (n = 19)	*t* or *u*	*p*
**Age**	72.37 ± 4.60	69.58 ± 4.51	1.89	0.067
**Sex**	Male: 6, Female: 13	Male: 6, Female: 13		
**Years of education**	10.21 ± 4.08	10.89 ± 3.93	−0.63	0.628
**GDS-K**	10.26 ± 2.00	2.21 ± 2.27	11.60	<0.001
**MMSE-KC**	27.89 ± 1.37	28.58 ± 0.77	−1.90	0.068
**Word-List Test (z-score)**				
**Learning**	0.37 ± 0.70	0.73 ± 0.84	−1.46	0.153
**Recall**	−0.45 ± 0.73	0.23 ± 0.88	−2.60	0.014
**Recognition**	−0.61 ± 0.83	0.48 ± 0.33	−4.13	<0.001
**eTIV (mm^3^)**	1.52 × 10^6^ ± 1.22 × 10^5^	1.52 × 10^6^ ± 1.61 × 10^5^	−0.05	0.964

GDS-K: Korean version of short Geriatric Depression Scale; MMSE-KC: Mini-Mental Status Examination in the Korean Version of the CERAD Assessment Packet; eTIV: estimated total intracranial volume.

**Table 2:** Volume of hippocampal subfields.

	Left Hippocampus						Right Hippocampus					
	Subclinical Depression	Control	F_group_	p_group_	ES	FDR	Subclinical Depression	Control	F_group_	p_group_	ES	FDR
**Parasubiculum**	59.44 ± 15.12	56.59 ± 13.17	0.41	0.526	0.011	0.570	51.91 ± 8.75	53.02 ± 7.06	0.05	0.827	0.006	0.827
**Presubiculum**	279.25 ± 40.09	297.26 ± 36.2	1.16	0.290	0.057	0.377	261.79 ± 31.84	284.42 ± 25.67	3.21	0.082	0.153	0.124
**Subiculum**	388.32 ± 46.81	435.28 ± 49.72	**6.21**	0.018	0.208	0.039	399.61 ± 55	444.59 ± 38.01	5.27	0.028	0.212	0.061
**CA1**	545.73 ± 62.86	614.26 ± 72.41	**6.41**	0.016	0.227	0.039	589.8 ± 68.66	662.84 ± 74.75	**6.36**	0.016	0.233	0.042
**CA3**	179.19 ± 24.03	208.56 ± 26.05	**9.44**	0.004	0.277	0.020	204.64 ± 26.82	226.4 ± 30.16	3.12	0.086	0.144	0.124
**CA4**	217.84 ± 25.93	241.02 ± 22.35	5.16	0.029	0.22	0.054	229.15 ± 24.38	257.3 ± 25.09	**7.78**	0.008	0.308	0.035
**GC-ML-DG**	248.06 ± 32.73	277.14 ± 30.52	4.37	0.044	0.218	0.064	261.91 ± 30.42	295.28 ± 32.66	**6.35**	0.016	0.279	0.042
**Molecular layer**	475.11 ± 52.46	535.01 ± 50.56	**8.77**	0.005	0.284	0.020	503.02 ± 59.13	564.59 ± 47.84	**8.08**	0.007	0.293	0.035
**HATA**	49.35 ± 12.17	52.72 ± 10.62	0.02	0.888	0.027	0.888	52.18 ± 9.39	55.98 ± 10.04	0.08	0.779	0.051	0.827
**Fimbria**	56.72 ± 24.1	67.7 ± 18.85	1.02	0.321	0.069	0.379	50.75 ± 24.61	63.95 ± 17.7	1.42	0.242	0.105	0.315
**Hippocampal tail**	478.7 ± 64.32	564.33 ± 55.91	**15.10**	<0.001	0.353	<0.001	526.88 ± 62.62	575.37 ± 49.44	4.11	0.050	0.179	0.093
**Hippocampal fissure**	146.91 ± 19.75	170.35 ± 35.74	4.93	0.033	0.149	0.054	166.39 ± 28.5	179.03 ± 37.85	1.11	0.300	0.036	0.355
**Whole hippocampus**	2976.78 ± 323.44	3350.84 ± 314.1	**8.52**	0.006	0.310	0.020	3130.97 ± 343.42	3484.41 ± 271.9	**7.79**	0.008	0.324	0.035

Hippocampal subfield volumes (mm^3^) are corrected for intracranial volume. Analysis of covariance with age as a covariate. F, *p* value of covariate can be found in the supplemental information. Bold: significant at *p* < 0.05 (corrected); ES: effect size, FDR: false discovery rate, CA: cornus ammonis, GC-ML-DG: granule cell and molecular layer of the dentate gyrus, HATA: hippocampus-amygdala-transition-area.

## Data Availability

Not applicable.

## Supplementary Materials

The following supporting information can be downloaded at: www.mdpi.com/article/10.3390/brainsci12030329/s1, Table S1. Statistical values for the analysis of covariance. Figure S1. Exploratory whole-brain connectometry analysis. Figure S2. Correlation between connectometry results and scores on the word-list test.
