# Supplementary material for: Hippocampal Subfields and White Matter Connectivity in Patients with Subclinical Geriatric Depression
Source: Brain Sci. 2022 Feb 28;12(3):329. doi: 10.3390/brainsci12030329 (PMC8946804; doi:10.3390/brainsci12030329)
Supplement: Supplementary file 1 [file brainsci-12-00329-s001.zip › Supplementry.pdf]

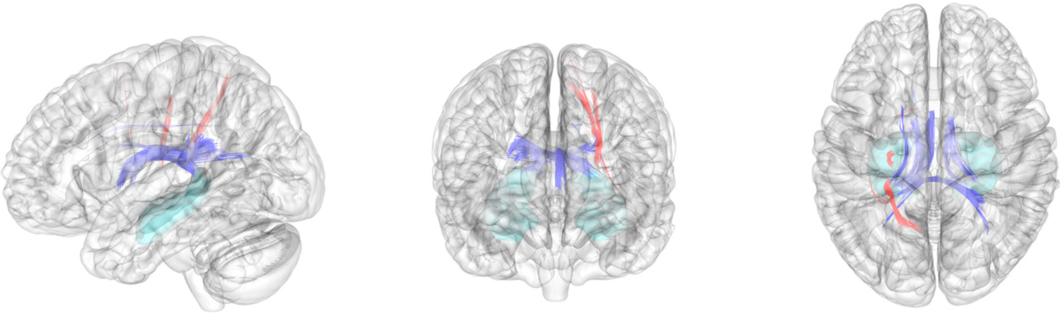

Figure S1. Exploratory whole-brain connectometry analysis. The majority of bilateral fornix fiber bundles exhibited decreased fractional anisotropy in the subclinical depression group. Fractional anisotropy in the left frontal parahippocampal cingulum, right superior thalamic radiation and tapetum, and fiber bundles of the forceps major of the corpus callosum were significantly lower in the subclinical depression group than in the control group (blue). Fractional anisotropy in the bundles of the left posterior thalamic radiation, left cortiospinal tract, tapetum of the corpus callosum, left parietal cortiocopontine tract, and left dentaorubrothalamic were higher in the subclinical depression group (red). The cyan shading indicates the hippocampus.

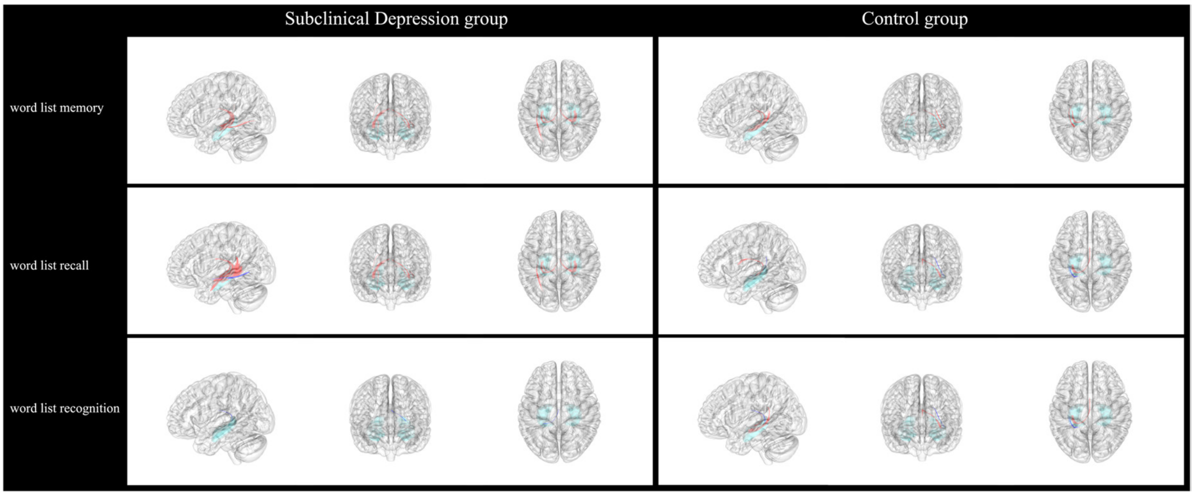

Figure S2. Correlation between connectometry results and scores on the verbal memory test. Fiber bundles exhibiting negative correlations with word-list scores following correction for multiple comparisons (false-discovery rate <0.05) are indicated in blue, while those exhibiting positive correlations with such scores are indicated in red. The cyan shading indicates the hippocampus. Note that the fiber bundle of the bilateral fornix exhibit a significant positive correlation with word-list memory and recall test results in both groups.

Table S1. Statistical values for the analysis of covariance.

	Age							
	Left Hippocampus				Right Hippocampus			
	**F_{Age}**	**P_{Age}**	**ES**	**FDR**	**F_{Age}**	**P_{Age}**	**ES**	**FDR**
Parasubiculum	0.04	0.847	0.011	0.847	4.41	0.072	0.006	0.043
Presubiculum	1.09	0.395	0.057	0.304	3.98	0.072	0.153	0.054
Subiculum	1.76	0.279	0.208	0.193	4.39	0.072	0.212	0.044
CA1	3.02	0.169	0.227	0.091	3.51	0.083	0.236	0.07
CA3	1.94	0.279	0.277	0.172	3.01	0.100	0.149	0.092
CA4	5.89	0.068	0.22	0.021	10.02	0.010	0.316	0.003
GC-ML-DG	8.77	0.046	0.218	0.005	10.46	0.010	0.289	0.003
Molecular layer	3.88	0.148	0.284	0.057	6.57	0.039	0.295	0.015
HATA	7.01	0.052	0.027	0.012	11.14	0.010	0.053	0.002
Fimbria	3.08	0.169	0.069	0.088	5.80	0.048	0.106	0.022
Hippocampal tail	0.91	0.410	0.353	0.347	3.95	0.072	0.179	0.055
Hippocampal fissure	0.21	0.701	0.149	0.647	0.01	0.911	0.036	0.911
Whole hippocampus	8.37	0.046	0.310	0.007	13.91	0.010	0.327	0.001

ES: effect size, FDR : false-discovery rate, CA: cornus ammonis, GC-ML-DG: granule cell and molecular layers of the dentate gyrus, HATA: hippocampus-amygdala-transition-area.
